# An Exploratory Model of Psychosocial Factors and Healthy Habits in University Students of Physical Education Depending on Gender

**DOI:** 10.3390/ijerph15112430

**Published:** 2018-11-01

**Authors:** Ramón Chacón-Cuberos, Félix Zurita-Ortega, Eva María Olmedo-Moreno, Rosario Padial-Ruz, Manuel Castro-Sánchez

**Affiliations:** 1 Department of Integrated Didactics, University of Huelva, 21007 Huelva, Spain; ramon.chacon@ddi.uhu.es; 2 Department of Didactics of Musical, Plastic and Corporal Expression, University of Granada, 18071 Granada, Spain; felixzo@ugr.es (F.Z.-O.); rpadial@ugr.es (R.P.-R.); 3 Department of Research Methods and Diagnosis in Education, University of Granada, 18071 Granada, Spain; emolmedo@ugr.es; 4 Department of Education. University of Almería, 04120 Almería, Spain

**Keywords:** motivational climate, Mediterranean diet, physical activity, substance abuse, maximum oxygen consumption, self-concept

## Abstract

(1) Background: Several researches have shown the relationship between healthy habits and physical and mental health. Thus, it is essential to study how some psychosocial factors can promote positive behaviours in university students, specifically in those who will be teachers of Physical Education. (2) Methods: This descriptive and cross-sectional research was conducted on 775 university students of Physical Education from Spain. This study aims to develop an explanatory model for the relationships between motivational climate, healthy habits (Mediterranean Diet (MD), Physical Activity (PA) and substance consumption) and some indicators of physical health (oxygen consumption (VO_2MAX_)) and mental health (self-concept) according to gender, using structural equations analysis. (3) Results: The motivational climate was positively associated with PA, showing a stronger relationship for ego-oriented climate in women. The adherence to MD showed a positive association with PA and self-concept, while it is negative for the consumption of tobacco in men. Likewise, PA was directly related to VO_2MAX_ with a higher regression weight for women. The ego-oriented motivational climate was negatively related to tobacco consumption in women. (4) Conclusions: Task-oriented goals are related to more positive and healthy behaviours such as PA, although it was no association was found with MD. Therefore, we can conclude the importance of promoting task-oriented goals in educational contexts linked to Physical Education in order to avoid negative behaviours.

## 1. Introduction

The vast majority of researches that study the university stage have been focused eminently on academic aspects despite the fact that this trend has changed in the last decade [1]. Psychosocial factors in young university students (such as their self-concept, motivation or body image), have been largely ignored. Thus, their association with healthy habits such as diet, physical activity or the absence of consumption of harmful substances, has gone unnoticed [2,3,4]. It is essential to study these variables in emerging adulthood, as they influence the lifestyle of young people and explain many of the negative behaviours that take place in this stage [5]. Therefore, the prevention and intervention of harmful behaviours, especially in those young people who will be future educators in the field of Physical Education, is a priority [6].

One of the main harmful habits existing in this period is the intake of junk food based on high contents of saturated fats, salt and sugar [7], making it necessary to become aware of its negative effects, as well as creating a willingness to reduce the intakes of this type of food [7,8]. Given this problem, different studies have highlighted the importance of promoting the Mediterranean diet (MD), which allows to prevent different diseases and to promote a good health status [9,10]. This dietary model is based on regular intake of food from the Mediterranean basin, highlighting a high consumption of foods rich in natural antioxidants such as legumes, fruits, nuts, vegetables, and olive oil, as well as a low consumption of fats, eggs and meat [9,11]. For this reason, it is interesting to promote this dietary model from an early age, in order to create healthy lifestyles and avoid future pathologies. In fact, several researches emphasize the beneficial nature of MD on public health, which is related to an improvement in the quality of life, as well as a minor risk of suffering cardiovascular diseases, infections or cancer—all reasons why its practice is of vital importance [11,12].

Nevertheless, not only is it important to follow an adequate diet, but also to follow an active lifestyle based on the practice of Physical Activity (PA) [13]. This term can be defined as any type of body movement that requires an energy expenditure to the skeletal muscles of the organism. In addition, a systematization of this movement with the aim of developing specific physiological adaptations will refer to the term “exercise” [14]. Several authors emphasize the relevance of practicing physical activity and sport in order to improve health, well-being and disease prevention [13,15,16]. In fact, the WHO [17] recommends performing at least 60 min of daily physical activity in children and young people. Among its benefits at a physiological level, the practice of PA involves a minor risk of suffering cardiovascular and coronary diseases, hypertension or type II diabetes, as well as an improvement in capillarization, bone mineral density, pulmonary perfusion, stroke volume or maximum oxygen consumption (VO_2MAX_) [18,19,20].

It has been demonstrated that at a cognitive level, following a healthy lifestyle decreases stress levels, improves memory and mood, favouring emotional stability, self-esteem and well-being [21,22]. Likewise, in the socio-affective sphere, physical-sporting practice can also be associated with the development of social skills and prosocial behaviours [23]. At this point, motivational factors can be related to healthy or unhealthy behaviours. This could be explained through the studies carried out by Deci & Ryan [24] and Balaguer et al. [25], which are based on the self-determination theory. These authors establish that those subjects who are extrinsically motivated, in the case of not achieving their objectives in the sports context, could use harmful behaviours linked to the consumption of substances due to the sensation of pleasure produced by their use and that will replace the generated frustration [24]. Therefore, it is essential to highlight two of the main psychosocial factors that are associated with healthy habits: Motivational climate in sport and self-concept [26,27].

The motivational climate is explained in achievement-goals theory, which has been widely used to give explanations of the motivations involved in sport practice [28]. This theory establishes that the objectives which people establish are related to their level of motivation in the practiced sport. Thus, individuals demonstrate two differentiated conceptions of ability. Task-oriented climate is associated with intrinsic and self-determined motivations for PA and the belief that skills are related to a learning process and the effort involved by people [29]. On the other hand, ego-oriented climate is associated with extrinsic motivations such as the need for getting better results than other people [30]. These motivational aspects will be determined by the strategies used by the different formative and socializing elements of the young people’s environment, such as teachers, coaches, parents or even friends [31]. Therefore, it is of vital importance to promote those guidelines that help to prevent unhealthy behaviours [5,32]. In fact, task-oriented motivational climates will relate to more positive and self-determined patterns of behaviour, while ego-oriented goals will be linked to extrinsic motivations and more negative behaviours [33].

Self-concept is a psychosocial factor which is associated with the image and perception that a person has of himself on a multidimensional level (physical, emotional, social, family and academic) and represents one of the most relevant psychosocial factors in emerging adulthood [34]. It has recently been shown that the self-concept of a subject can be related to the well-being of this person [35]. Thus, specific levels of self-concept could be promoters of positive behaviours such as healthy habits or positive emotional states. In addition, low levels of self-concept could be a risk factor of harmful habits such as sedentary lifestyle, poor quality in the diet, consumption of harmful substances or poor academic performance among others [36]. It has been shown that habits such as good nutrition or the practice of PA will help to increase the levels of this psychosocial factor [37]. Therefore, it is essential to promote certain motivational climates that help to shape healthy habits, which will ultimately improve self-concept.

Therefore, the present study develops a structural equation model in which the motivational climate in sport is included as an exogenous variable that infers various healthy habits (tobacco and alcohol consumption, PA and MD), which act as endogenous variables that receive the effect of the motivational climate. Following the premises exposed in the theoretical framework of this research, VO_2mMAX_ and self-concept are included as indicators of physical and mental health. These variables are also included as endogenous variables that receive the direct effect of PA, MD and the consumption of alcohol and tobacco, and the negative effect of the motivational climate. In addition, it has been shown that the association between these variables can vary with gender, which is why a multi-group analysis is carried out.

For this reason, the following objectives are set in the present research: (a) to develop an explanatory model about the relationships between motivational climate, healthy habits (PA, MD and substance abuse) and health indicators (VO_2MAX_ and self-concept); (b) to contrast the structural model developed according to gender through multi-group analysis.

## 2. Materials and Methods

### 2.1. Subjects and Design

A non-experimental, cross-sectional and ex post-facto study was carried out with a single measurement in a single group. The sample consisted of 775 university students from the eight provinces of Andalucia (Spain), with a gender representation of 58.7% (*n* = 455) for men and a 41.3% (*n* = 320) for women. The mean age was 22.22 ± 3.76 (range: 20 to 29 years old) and the criteria for sample selection was to study the mention of Physical Education in the Degree of Primary Education in any of the eight provinces of Andalucia. The students enrolled in PE degrees in Andalucia were 1167 for the academic year 2016/2017, according to the data provided by the different universities. For this population, 775 students were selected using simple random sampling following the statements established by Merino-Marban et al. [38] for natural groups.

### 2.2. Measures

Motivational climate was evaluated through the Perceived Motivational Climate in Sport Questionnaire (PMCSQ-2, [39]), using the Spanish version validated by González-Cutre et al. [40]. This scale is composed of 33 five-point items ranging from 1 to 5 (1 = Strongly Disagree; 5 = Strongly Agree). This questionnaire is comprised of two dimensions (Task-climate and Ego-climate), each containing three subscales: Cooperative Learning, Effort/Improvement, and Important Role; and Punishment for Mistakes, Unequal Recognition, and Member Rivalry, respectively. For this scale, the internal consistency got an acceptable value of Cronbach’s alpha (α = 0.82).

Alcohol consumption was assessed with the Alcohol Use Disorders Identification Test (AUDIT, [41]), which was translated to Spanish by Rubio [42]. This 10-item scale is evaluated using a five-point Likert scale where 0 is “Never” and 4 is “Daily” for the first eight items. In addition, the last two items are evaluated using a three-point Likert scale which generates a point score of 0, 2 or 4 points. Responses are summed to produce an overall score relating to alcohol consumption. This instrument got an acceptable Cronbach’s alpha of α = 0.75.

Levels of PA were evaluated using the Physical Activity Questionnaire for Adolescents (PAQ-A; [43]), which was translated to Spanish by Martínez-Gómez et al. [44]. This scale is used in order to evaluate the level of practice of PA during the last week. This instrument allows to get a summation from 10 items which are scored through a five-point Likert scale where 0 is “Never” and 4 is “Always”. In the present study, this scale obtained an acceptable Cronbach’s alpha of α = 0.80.

The level of adherence to MD was assessed through The Evaluation of the Mediterranean Diet Index (KIDMED) [45]. This scale is composed of 16 dichotomous items which can be answered with yes or no (there are 12 positive items and 4 negative items). Finally, these items conform an overall punctuation which lies in the range of −4 to 12. This scale obtained an acceptable internal consistency, showing a Cronbach’s alpha of α = 0.83.

The Spanish adaptation by Villareal-González [46] of The Fagerström Test for Nicotine Dependence (FTND, [47]) was employed in order to evaluate tobacco consumption. This instrument evaluates the number or amount of cigarettes, impulse to smoke and nicotine dependency. It includes six questions. The first four are dichotomous (0 = No and 1 = Yes), and the other two follow a four-option Likert-type scale (0 = Never and 3 = Always). The sum of items ranges between 0 and 10, establishing the level of dependence to nicotine. This study got an excellent Cronbach’s alpha of α = 0.96.

The “Meter Shuttle Run Test (20mSRT) [48] was used for measuring the VO_2MAX_. This test consists of running repeatedly a distance of 20 m with an incremental speed, starting with an initial speed of 8 km/h which increase 0.5 km/h per minute. The VO_2MAX_ is indirectly calculated through the speed achieved in the last period. For this aim, the following formula is used: VO_2MAX_ (mL/min/kg) = (6 × FA) − 27.4 [49,50].

Finally, self-concept was assessed through the Five-Factor Self-Concept Questionnaire (AF-5) [51]. This test includes 30 items which are rated on a five-point Likert scale (1 = Never; 5 = Always). The negative items are inverted and a summation of the score obtained in each one is made in order to obtain a total score for the self-concept. The AF-5 showed an acceptable Cronbach ‘s alpha with a value of α = 0.86.

### 2.3. Procedure

First, the participants’ collaboration was requested using an informative document prepared from the Corporal Area of the University of Granada. It was administered to the university students who attended the Mention of Physical Education in the Degree of Primary Education in the eight Andalusian provinces through the departments of the different universities. The nature and objectives of the research to be performed were detailed in this document. Moreover, the informed consent to participate in this investigation was requested.

Second, respondents were instructed for the data collection process. All scales were applied in a normal teaching lesson at the university without providing incentives for that aim. Furthermore, a research assistant was present in order to help those respondents who had any difficulty. It is important to establish that the Ethics Committee of the University of Granada approved this research (462/CEIH/2017). In addition, this study followed the Ethical principles of the Declaration of Helsinki for medical research.

### 2.4. Statistical Analysis

**Figure 1:**
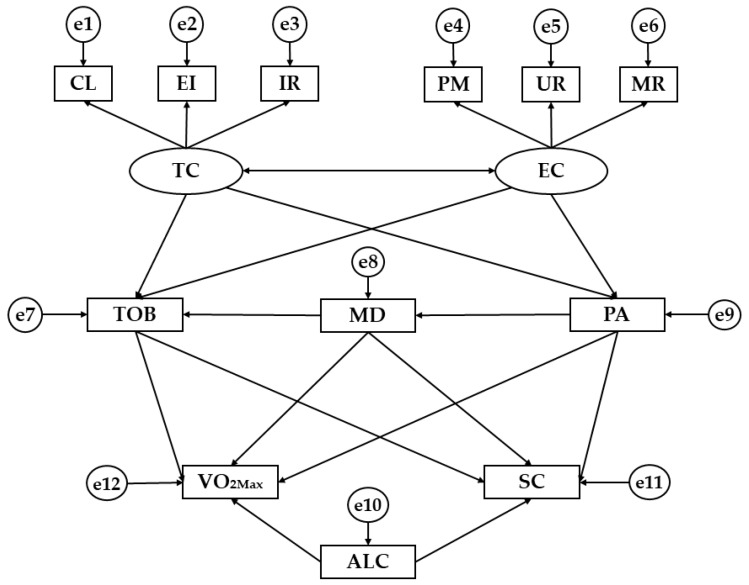
Structural Equation Model. Note: TC, Task Climate; EC, Ego Climate; CL, Cooperative Learning; EI, Effort/Improvement; IR, Important Role; PM, Punishment for Mistakes; UR, Unequal Recognition; MR, Member Rivalry; TOB, Tobacco; MD, Mediterranean Diet; PA, Physical Activity; ALC, Alcohol; VO_2MAX_, Maximum oxygen consumption (mL/min/kg); SC; Self-concept.

Descriptive analysis was carried out using the software SPSS^®^ version 22.0 (IBM Corp., Armonk, NY, USA), while the Structural Equation Model (SEM) was developed through the software AMOS^®^ version 22.0 (IBM Corp., Armonk, NY, USA). For theses analysis, the level of significance was set at 0.05 using the Pearson Chi-square test. In addition, the Cronbach’s Alpha coefficient was used to establish the internal reliability of the scales, fixed at 95.5%. The SEM developed is shown in Figure 1.

The Structural Equation Model shown in Figure 1 is composed of two latent variables (ovals) and 12 observed variables (squares). In addition, each observed variable is associated with an error term (circles). Task Climate (TC) and Ego Climate (EC) represents the latent variables which are exogenous. These two latent variables were inferred by the following six observed variables: Cooperative Learning (CL), Important Role (RI) and Effort/Improvement (EI) for Task Climate (TC), and Unequal Recognition (UR), Member Rivalry (MR) and Punishment for Mistakes (PM) for Ego Climate (EC). Other latent variables were Tobacco consumption (TOB), adherence to Mediterranean diet (MD), Physical Activity (PA), Alcohol consumption (ALC), VO_2MAX_, and Self-Concept (SC), which act as endogenous variables. Furthermore, the unidirectional arrows associate with the observed variables showing the influence between them, while bi-directional arrows relate to the latent variables. It is important to highlight that these arrows can be interpreted as multivariate regression coefficients.

Model fit was examined in order to verify the reliability of the SEM developed through several indices [52] and the method of maximum likelihood (ML) was employed to estimate associations between constructs. Chi-squared analysis show a good fit for non-significant *p*-values. In addition, other indices are employed such as Normalized Fit Index (NFI), Increase Fit Index (IFI) and Comparative Fit Index (CFI). Values higher than 0.90 indicate an acceptable fit, and values higher than 0.95 show an excellent fit. Finally, the Root Mean Square Error of Approximation (RMSEA) set an acceptable fit for values below 0.08, and an excellent fit for values below 0.05.

## 3. Results

The indices analyzed for this SEM suggest a good fit. First, the chi-square showed a significant value (χ^2^ = 462.83; df = 78; *p* < 0.001). Nevertheless, it is important to establish that this index is really sensitive to sample size, and it is essential to take into consideration other standardized indices [52]. The NFI got a value of 0.91, the CFI showed a value of 0.92 and the IFI yielded a value of 0.92, showing an acceptable fit for the model. Moreover, the RMSEA showed a value of 0.08, revealing an acceptable fit for this index [52]. Thus, these values suggest that the SEM fits the empirical data properly.

**Figure 2:**
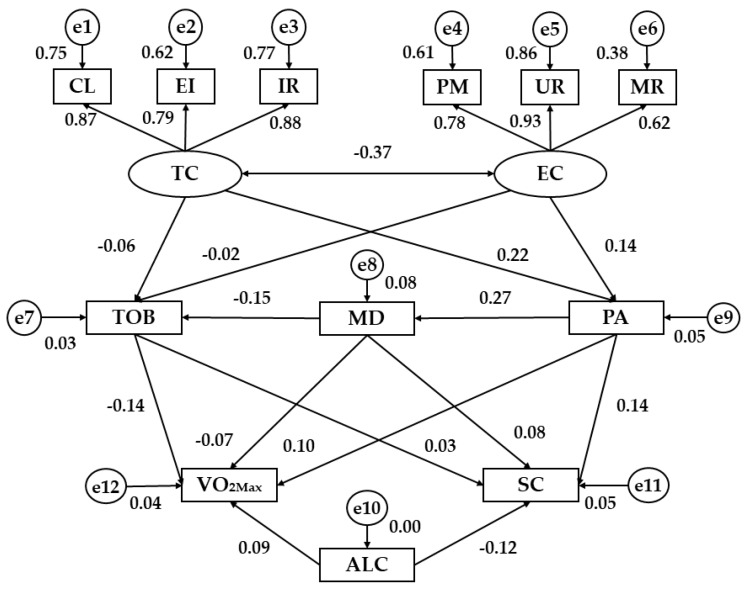
Structural equation model for men. Note: TC, Task Climate; EC, Ego Climate; CL, Cooperative Learning; EI, Effort/Improvement; IR, Important Role; PM, Punishment for Mistakes; UR, Unequal Recognition; MR, Member Rivalry; TOB, Tobacco; MD, Mediterranean Diet; PA, Physical Activity; ALC, Alcohol; VO_2MAX_, Maximum oxygen consumption (mL/min/kg); SC; Self-concept.

Table 1 and Figure 2 show the regression weights of the structural model for men. Analyzing the motivational climate in sport as an exogenous variable, a negative association was found between task climate and ego climate (*p* < 0.001; b = −0.37). Furthermore, the three observed variables which define these two latent variables were directly associated with their respective dimension (*p* < 0.001). The strongest indicator for task climate was an important role (b = 0.88) and for ego climate was unequal recognition (b = 0.93). The indicators with the lowest regression weight were effort/improvement (b = 0.79) and member rivalry (b = 0.62), respectively.

Addressing the relationship between motivational climate and healthy habits, PA was associated with task climate (*p* < 0.001; b = 0.22) and ego climate (*p* < 0.01; b = 0.14) in men, showing a positive relationship in both cases. Nevertheless, the MD and tobacco consumption were not related to the dimensions established for motivational climate. In addition, there was a positive relationship between MD and PA (*p* < 0.001; b = 0.27) and a negative association between MD and tobacco consumption (*p* < 0.01; b = −0.15).

Finally, the second level of the model analyzes the relationship between healthy habits and indicators of health status. PA was directly related to VO_2MAX_ (*p* < 0.05; b = 0.10) and self-concept (*p* < 0.01; b = 0.14), as well as MD was positively associated with self-concept (*p* < 0.05; b = 0.08), but not with VO_2MAX_. Results showed a positive relationship between alcohol and VO_2MAX_ (*p* < 0.05; b = 0.09). Tobacco consumption was negatively associated with VO_2MAX_ (*p* < 0.01; b = −0.14) and alcohol consumption was inversely related to self-concept (*p* < 0.01; b = −0.12). In addition, tobacco consumption was not associated with self-concept.

**Figure 3:**
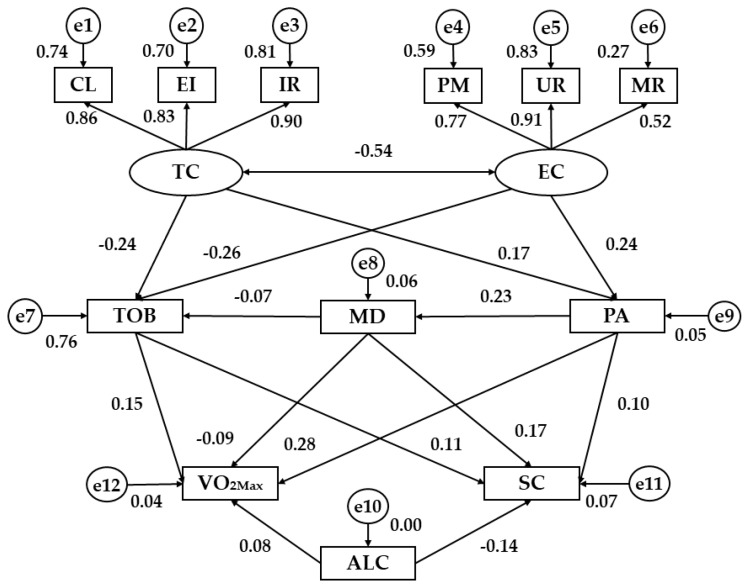
Structural equation model for women. Note: TC, Task Climate; EC, Ego Climate; CL, Cooperative Learning; EI, Effort/Improvement; IR, Important Role; PM, Punishment for Mistakes; UR, Unequal Recognition; MR, Member Rivalry; TOB, Tobacco; MD, Mediterranean Diet; PA, Physical Activity; ALC, Alcohol; VO_2MAX_, Maximum oxygen consumption (mL/min/kg); SC; Self-concept.

Table 2 and Figure 3 show the regression weights for women. Analyzing the motivational climate in sport as an exogenous variable, a negative association was found between task climate and ego climate (*p* < 0.001; b = −0.54). Moreover, the three observed variables which are related to the two latent variables were directly associated with their respective dimension (*p* < 0.001). The strongest indicator for task climate was important role (b = 0.90) and for ego climate was unequal recognition (b = 0.91). The indicators with the lowest regression weight were effort/improvement (b = 0.83) and member rivalry (b = 0.52), respectively.

Addressing the relationship between motivational climate and healthy habits, PA was associated with task climate (*p* < 0.001; b = 0.17) and ego climate (*p* < 0.01; b = 0.24), showing a positive relationship in both cases. Nevertheless, the MD was not related to the dimensions established for motivational climate. Tobacco consumption showed a negative relationship with task climate (*p* < 0.001; b = −0.24) and ego climate (*p* < 0.001; b = −0.26). In addition, there was a positive relationship between MD and PA (*p* < 0.001; b = 0.23), while MD was not associated with tobacco consumption.

Finally, the second level of the model analyzes the relationship between healthy habits and indicators of health status. PA was directly related to VO_2MAX_ (*p* < 0.001; b = 0.28) but there were no associations with self-concept. On the contrary, MD was directly related to self-concept (*p* < 0.01; b = 0.17), but not with VO_2MAX_. Results did not show relationships between alcohol and VO_2MAX_. Tobacco consumption was directly associated with VO_2MAX_ (*p* < 0.01; b = 0.15) and alcohol consumption was negatively associated with self-concept (*p* < 0.01; b = −0.14). In addition, tobacco consumption was directly related to self-concept in women (*p* = 0.05; b = 0.11).

## 4. Discussion

The present research was conducted with university students of Physical Education given the interest that this sample presents as emerging adults who will train and educate children in the field of health in the future. The main objective of this research was to develop a structural model that explains the relationship between the motivational climate in sport and healthy habits. Moreover, a second level is added to the model, in which two indicators of the level of physical health (VO_2MAX_) and mental health (self-concept) appear. This is done in order to check the direct effect of healthy habits on these variables and the negative effect of the motivational climate in them. In addition, a multi-group analysis is developed in order to compare the relationships between these variables according to the gender of the students with the aim to provide a view closer to reality. In this way, some researches of similar line are those developed by Chacón et al. [5], Hardcastle et al. [33], Varela-Mato et al. [53], Ganasegeran et al. [54] and Chacón-Cuberos et al. [55].

First, it is important to point out that the motivational climate in sport represents the exogenous variable of the structural model, and that it will have an effect on the other endogenous variables (healthy habits). In this way, an inverse association could be observed between the task-oriented climate and the ego-oriented climate, which acquired greater strength for women. Although these findings have been previously demonstrated given the greater tendency of women to position themselves for task-oriented motivational climates [31,56], it should be noted that in this research, a difference was not as pronounced as in others [5,57]. This is due to the fact that both sexes are academically and professionally engaged in sports, so their tendency towards competition is higher and shows elevated values in both ego climates. A clear example of this is that the most influential indicators for both motivational orientations are the same, although unequal recognition and rivalry between members is higher in men [32,33].

Considering the relationship between motivational climate and different healthy habits, PA was related to both motivational climates, obtaining a greater correlation strength for task climate in men and for ego-oriented climate in women. It could indicate that women practice sport, being motivated by extrinsic goals such as competition or achieving physical attractiveness [29,58], while men pursue more self-determined motivations such as enjoying or learning new skills in sports practice [59]. Nevertheless, it should be noted that in the case of the relationship between task climate and PA in women, the level of significance is set at *p* < 0.05, which means that statistical differences are shown for relatively low regression weights. Furthermore, MD was not related to the motivational climate for both groups, and this relationship was deleted from the path model. These results are contrary to those obtained by Chacón et al. [5] or Leyton et al. [60]. This may be due to the fact that respondents follow a diet of good quality, since it influences sports performance and all participants are athletes. In spite of this, it has been demonstrated that in non-sporting contexts, the task-oriented motivational climate is linked to a better quality of the diet, since the subjects who are intrinsically motivated will not suffer stress and frustration in the face of sports defeats, which would lead them to make unhealthy meals as a mean to generate satisfaction [5,60]. Likewise, motivational climate was not associated with alcohol and tobacco in men, while tobacco consumption has a negative relationship with both motivational climates in women. Thus, it could be concluded that the promotion of physical-sport practice could help reduce this type of harmful habit [5,33,53].

Subsequently, the relationships between healthy habits were analysed. According adherence to MD, findings reveal a direct relationship between this variable and the practice of PA, which was higher in women. This could be explained by the greater association found in this sex for the ego-oriented motivational climate, which would imply a greater care of the diet for the improvement of the physical fitness for competition and physical attractiveness [36,61]. In addition, MD was inversely associated with tobacco consumption in men, which could demonstrate that to follow an appropriate diet could help to prevent the consumption of this substance, as shown by Phull et al. [62]. In this line, adherence to MD was positively related to self-concept, especially in women. Zurita-Ortega et al. [63] show how following a healthy diet helps in the improvement of physical and academic self-concept by improving body composition and school performance.

Analysing the second level of the model—the association between healthy habits and indicators of health status—it was found that PA showed a positive and direct relationship with VO_2MAX_ and self-concept. The first relationship should also be interpreted with caution in men, since a low level of significance was obtained for the reasons described above. Nevertheless, the relationship between PA and VO_2MAX_ acquired greater strength in women. It is evident that physical exercise will produce various physiological benefits such as the improvement of cardiac output, the exchange of pulmonary gases or muscle capillarization, which will increase the maximum oxygen consumption [13,19,20]. Moreover, PA will also improve body composition, which will directly affect the physical dimension of self-concept [26,64]. It should be noted that MD was not related to VO_2MAX_, which could be justified by the greater dependence of this indicator on sport habits and training intensity parameters. In addition, it has been demonstrated that a high adherence to MD is linked to a better health state, but not with the adaptations linked to a higher VO_2MAX_ such as lung volume or better perfusion of respiratory gases [19,61]. Finally, PA did not reveal statistically significant associations with respect to alcohol consumption. This could be due to the expansion of this substance among young adults [65,66], which means that conclusive results cannot be obtained in this regard.

Analysing the consumption of harmful substances, it should be highlighted that alcohol consumption was not related to VO_2MAX_ in women. Nevertheless, a negative relationship was observed between tobacco consumption and VO_2MAX_ for men as well as a positive association for women. These results may seem contradictory given that tobacco consumption decreases lung function in addition to increasing the risk of suffering from chronic obstructive pulmonary disease [67]. Nevertheless, these findings could be justified by the positive effect of the practice of PA in women, decreasing the negative effect of tobacco on VO_2MAX_. With respect to self-concept, it has been proven that alcohol intake is negatively related to it, since Chacón et al. [66] and Lindgren et al. [68] establish the negative relationship of this substance with its familiar, academic and physical dimensions. In these studies, only positive relationships were found with the social dimension of self-concept. In fact, this premise could justify the direct relationship given between the consumption of tobacco and the self-concept of women in the present research, even though it is a relationship with a low level of significance.

Finally, it is essential to highlight the limitations of this research. The first of these lies in the design of the research, since cross-sectional studies do not allow to establish causal relationships. Nevertheless, the structural model developed allows knowing the associations given between the variables in an effective way. Another limitation is related to the values of significance given for *p*-value. The structural equation model considers three levels of significance (*p* < 0.05, *p* < 0.01, *p* < 0.001). This means that having a high sample, significant relationships are obtained at *p* < 0.05 for several variables, making them show relatively low regression weights. As a solution, the significance level could be set to *p* < 0.001 for future studies. Nevertheless, it can be established that the sample size is adequate for the model developed since the method of maximum likelihood in the analysis of covariance is employed. In addition, a valid coefficient is obtained for the root Mean Square Error of Approximation and the standard error bias for parameters does not exceed 5% [69]. Finally, the variables used could represent another limitation, since only VO_2MAX_ was used to assess health status. Thus, as future perspectives there is a need to replicate this model in other groups by expanding the sample, as well as the inclusion of other indicators of health, such as the percentage of lean and fat mass or hand grip strength. It would also be interesting to include other psychosocial factors related to these habits and mental health, such as self-esteem or body image.

## 5. Conclusions

As main conclusions, it can be noted that there is a positive association between MD and PA in both genders, being slightly higher in women. There is a direct relationship between MD and self-concept, which is higher in women despite the fact that MD is not related to the motivational climate in sport and VO_2MAX_ for men and women. In addition, PA was related to both motivational climates, obtaining a greater correlation strength for the task climate in men and the ego climate in women. Likewise, PA was positively associated with VO_2MAX_ in both sexes and with self-concept in males, showing its beneficial effects. Finally, alcohol was negatively related to self-concept in both sexes, while smoking was positively related to self-concept in women due to social issues. It can be established that following an active lifestyle and good nutrition is related to a better state of physical and mental health. Although the motivational climate was not related to MD, it was associated with PA, which was directly related to the diet followed. Thus, the importance of developing task-oriented motivational climates in order to promote healthier habits and to avoid negative behaviour in university students should be highlighted.

## Figures and Tables

**Table 1:** Weights and standardized regression weights for men.

Relationship between Variables			Regression Weights				S.R.W
			Estimate	S.E.	C.R.	*p*	Estimate
PA	←	TC	3.134	0.756	4.146	***	0.22
PA	←	EC	1.887	0.723	2.609	**	0.14
MD	←	PA	0.082	0.014	5.750	***	0.27
TOB	←	TC	−0.288	0.281	−1.025	0.305	−0.06
TOB	←	EC	−0.076	0.271	−0.279	0.780	−0.02
TOB	←	MD	−0.184	0.057	−3.258	**	−0.15
VO_2MAX_	←	MD	−21.522	14.720	−1.462	0.144	−0.07
VO_2MAX_	←	PA	9.178	4.492	2.043	*	0.10
VO_2MAX_	←	TOB	−34.737	11.763	−2.953	**	−0.14
VO_2MAX_	←	ALC	17.114	8.771	1.951	*	0.09
CL	←	TC	1.000	-	-	***	0.87
EI	←	TC	0.760	0.039	19.344	***	0.79
IR	←	TC	1.019	0.047	21.582	***	0.88
PM	←	EC	1.000	-	-	***	0.78
UR	←	EC	1.316	0.082	16.040	***	0.93
MR	←	EC	0.930	0.070	13.228	***	0.62
SC	←	TOB	0.004	0.006	0.730	0.466	0.03
SC	←	ALC	−0.011	0.004	−2.681	**	−0.12
SC	←	PA	0.006	0.002	2.901	**	0.14
SC	←	MD	0.012	0.007	1.687	*	0.08
EC	↔	TC	−0.145	0.023	−6.286	***	−0.37

Note 1: S.R.W., Standardized Regression Weights; S.E., Estimation of Error; C.R., Critical Ratio. Note 2: TC, Task Climate; EC, Ego Climate; CL, Cooperative Learning; EI, Effort/Improvement; IR, Important Role; PM, Punishment for Mistakes; UR, Unequal Recognition; MR, Member Rivalry; TOB, Tobacco; MD, Mediterranean Diet; PA, Physical Activity; ALC, Alcohol; VO_2MAX_, Maximum oxygen consumption (mL/min/kg); SC; Self-concept. Note 3: * *p* < 0.05; ** *p* < 0.01; *** *p* < 0.001. Note 4: ←, relationships between observed variables; ↔, relationships between latent variables.

**Table 2:** Weights and standardized regression weights for women.

Relationship between Variables			Regression Weights				S.R.W
			Estimate	S.E.	C.R.	*p*	Estimate
PA	←	TC	2.053	0.900	2.280	*	0.17
PA	←	EC	3.208	0.987	3.249	**	0.24
MD	←	PA	0.069	0.017	4.083	***	0.23
TOB	←	TC	−1.158	0.351	−3.295	***	−0.24
TOB	←	EC	−1.364	0.385	−3.542	***	−0.26
TOB	←	MD	−0.086	0.071	−1.213	0.225	−0.07
VO_2MAX_	←	MD	−19.462	11.317	−1.720	0.085	−0.09
VO_2MAX_	←	PA	17.183	3.421	5.022	***	0.28
VO_2MAX_	←	TOB	24.550	8.539	2.875	**	0.15
VO_2MAX_	←	ALC	10.496	7.272	1.443	0.149	0.08
CL	←	TC	1.000	-	-	***	0.86
EI	←	TC	0.878	0.048	18.377	***	0.83
IR	←	TC	1.072	0.053	20.123	***	0.90
PM	←	EC	1.000	-	-	***	0.77
UR	←	EC	1.294	0.099	13.082	***	0.91
MR	←	EC	0.769	0.086	8.974	***	0.52
SC	←	TOB	0.014	0.007	2.027	*	0.11
SC	←	ALC	−0.016	0.006	−2.670	**	−0.14
SC	←	PA	0.005	0.003	1.608	0.108	0.10
SC	←	MD	0.029	0.009	3.125	**	0.17
EC	↔	TC	−0.217	0.031	−6.902	***	−0.54

Note 1: S.R.W., Standardized Regression Weights; S.E., Estimation of Error; C.R., Critical Ratio. Note 2: TC, Task Climate; EC, Ego Climate; CL, Cooperative Learning; EI, Effort/Improvement; IR, Important Role; PM, Punishment for Mistakes; UR, Unequal Recognition; MR, Member Rivalry; TOB, Tobacco; MD, Mediterranean Diet; PA, Physical Activity; ALC, Alcohol; *V*O_2MAX_, Maximum oxygen consumption (mL/min/kg); SC; Self-concept. Note 3: * *p* < 0.05; ** *p* < 0.01; *** *p* < 0.001. Note 4: ←, relationships between observed variables; ↔, relationships between latent variables.
